# (*Z*)-Methyl 2-[(2-eth­oxy-6-formyl­phen­oxy)meth­yl]-3-(4-ethyl­phen­yl)acrylate

**DOI:** 10.1107/S1600536811046587

**Published:** 2011-11-09

**Authors:** Rajeswari Gangadharan, K. Sethusankar, Raman Selvakumar, Manickam Bakthadoss

**Affiliations:** a Department of Physics, Ethiraj College for Women (Autonomous), Chennai 600 008, India; b Department of Physics, RKM Vivekananda College (Autonomous), Chennai 600 004, India; c Department of Organic Chemistry, University of Madras, Maraimalai Campus, Chennai 600 025, India

## Abstract

The title compound, C_22_H_24_O_5_, consists of two substituted benzene rings linked by an ethyl acrylate group. The dihedral angle between the two benzene rings is 58.39 (7)°. The crystal packing is governed by two C—H⋯O inter­actions, one of which forms centrosymmetric dimers with a graph-set descriptor of *R*_2_^2^(18).

## Related literature

For applications of acrylate derivatives, see: Xiao *et al.* (2008 ▶). For a related structure, see: Gong *et al.* (2008 ▶). For graph-set notation, see: Bernstein *et al.* (1995 ▶).
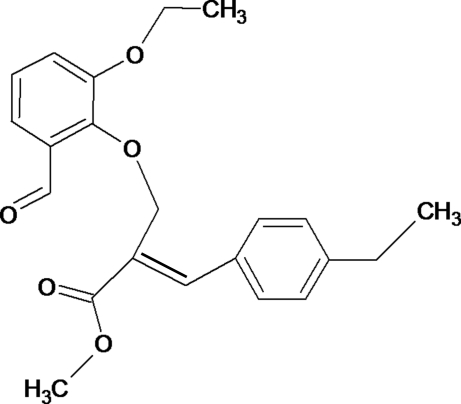


## Experimental

### 

#### Crystal data

C_22_H_24_O_5_
*M* *_r_* = 368.41
Triclinic, 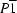

*a* = 9.6746 (3) Å
*b* = 9.9435 (3) Å
*c* = 10.7935 (3) Å
α = 77.595 (1)°
β = 85.433 (2)°
γ = 76.752 (1)°
*V* = 986.59 (5) Å^3^
*Z* = 2
Mo *K*α radiation
μ = 0.09 mm^−1^
*T* = 293 K
0.30 × 0.25 × 0.20 mm

#### Data collection

Bruker SMART APEXII area-detector diffractometer
25506 measured reflections
6646 independent reflections
4400 reflections with *I* > 2σ(*I*)
*R*_int_ = 0.025

#### Refinement

*R*[*F* ^2^ > 2σ(*F* ^2^)] = 0.051
*wR*(*F* ^2^) = 0.164
*S* = 1.02
6646 reflections
247 parameters
H-atom parameters constrained
Δρ_max_ = 0.25 e Å^−3^
Δρ_min_ = −0.21 e Å^−3^

### 

Data collection: *APEX2* (Bruker, 2008 ▶); cell refinement: *SAINT* (Bruker, 2008 ▶); data reduction: *SAINT*; program(s) used to solve structure: *SHELXS97* (Sheldrick, 2008 ▶); program(s) used to refine structure: *SHELXL97* (Sheldrick, 2008 ▶); molecular graphics: *ORTEP-3* (Farrugia, 1997 ▶); software used to prepare material for publication: *SHELXL97* and *PLATON* (Spek, 2009 ▶).

## Supplementary Material

Crystal structure: contains datablock(s) global, I. DOI: 10.1107/S1600536811046587/pv2468sup1.cif

Structure factors: contains datablock(s) I. DOI: 10.1107/S1600536811046587/pv2468Isup2.hkl

Supplementary material file. DOI: 10.1107/S1600536811046587/pv2468Isup3.cml

Additional supplementary materials:  crystallographic information; 3D view; checkCIF report

## Figures and Tables

**Table 1:** Hydrogen-bond geometry (Å, °)

*D*—H⋯*A*	*D*—H	H⋯*A*	*D*⋯*A*	*D*—H⋯*A*
C7—H7⋯O5^i^	0.93	2.58	3.368 (2)	143
C17—H17⋯O1^ii^	0.93	2.56	3.454 (2)	161

Symmetry codes: (i) 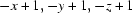; (ii) 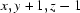.

**Fig. 1.**
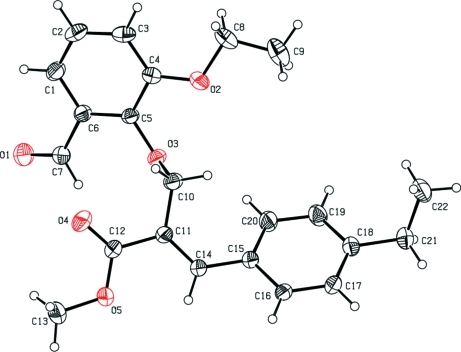
The molecular structure of the title compound with the atom numbering scheme, displacement ellipsoids are drawn at 30% probability level. H atoms are drawn as small spheres of arbitary radii.

**Fig. 2.**
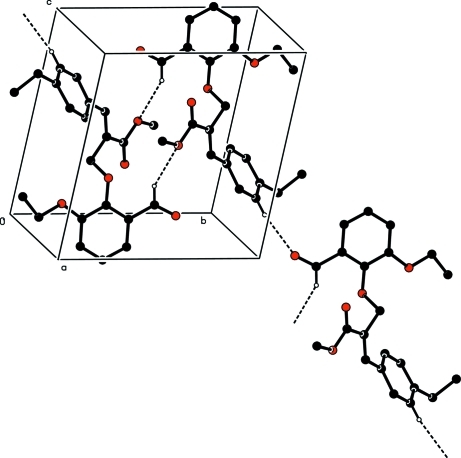
Part of crystal structure of the title compound, showing the formation of R22(18) dimers viewed along the a-axis. Dashed lines indicates C—H···O intermolecular interactions.

## supplementary crystallographic information

### Comment

Phenyl acrylates show considerable antibacterial activities against Staphylococcus aureus (Xiao *et al.*, 2008).

In the title compound (Fig. 1), the methyl acrylate group is planar and forms dihedral angles 33.43 (4)° and 41.71 (4)° with the two phenyl rings (C1–C6) and (C15–C20), respectively. The inter planar angle between the two phenyl rings is 58.39 (7)°. The title compound exhibits structural similarities with the already reported related structure (Gong *et al.*, 2008).

The crystal packing is governed by two C—H···O interactions, one of which forms centrosymmetric dimers with graph set descriptor of *R*^2^_2_(18) (Bernstein *et al.*1995) (Fig. 2 and Table 1)

### Experimental

A solution of 3-ethoxysalicylaldehyde (3.54 mmol, 0.58 g) and potassium carbonate (3.89 mmol, 0.53 g) in acetonitrile (10 ml) was stirred for 15 minutes at room temperature. To this solution, (*Z*)-methyl 2-(bromomethyl)-3-(4-ethylphenyl)acrylate (3.54 mmol, 1 g) was added dropwise. After the completion of the reaction as indicated by TLC, acetonitrile was evaporated. Ethylacetate (15 ml) and water (15 ml) were added to the crude mass and extracted. The organic layer was dried over anhydrous sodium sulfate. Removal of solvent led to the crude product which was purified through pad of silica gel (100–200 mesh) using ethylacetate and hexanes (1:9) as solvents. The pure title compound was obtained as a colorless solid (1.05 g, 81%). Recrystallization was carried out using ethylacetate as solvent.

### Refinement

The hydrogen atoms were placed in calculated positions with C—H = 0.93 to 0.97 Å and refined in the riding model with isotropic displacement parameters: *U*_iso_(H) = 1.5*U*_eq_(C)for methyl group and *U*_iso_(H) = 1.2*U*_eq_(C)for other groups.

### Crystal data

**Table:**

C_22_H_24_O_5_	*Z* = 2
*M**_r_* = 368.41	*F*(000) = 392
Triclinic, *P*1	*D*_x_ = 1.240 Mg m^−^^3^
Hall symbol: -P 1	Mo *K*α radiation, λ = 0.71073 Å
*a* = 9.6746 (3) Å	Cell parameters from 6646 reflections
*b* = 9.9435 (3) Å	θ = 1.9–31.7°
*c* = 10.7935 (3) Å	µ = 0.09 mm^−^^1^
α = 77.595 (1)°	*T* = 293 K
β = 85.433 (2)°	Block, colorless
γ = 76.752 (1)°	0.30 × 0.25 × 0.20 mm
*V* = 986.59 (5) Å^3^	

### Data collection

**Table:**

Bruker SMART APEXII area-detector diffractometer	4400 reflections with *I* > 2σ(*I*)
Radiation source: fine-focus sealed tube	*R*_int_ = 0.025
graphite	θ_max_ = 31.7°, θ_min_ = 1.9°
ω and φ scans	*h* = −13→14
25506 measured reflections	*k* = −14→14
6646 independent reflections	*l* = −15→15

### Refinement

**Table:**

Refinement on *F*^2^	Primary atom site location: structure-invariant direct methods
Least-squares matrix: full	Secondary atom site location: difference Fourier map
*R*[*F*^2^ > 2σ(*F*^2^)] = 0.051	Hydrogen site location: inferred from neighbouring sites
*wR*(*F*^2^) = 0.164	H-atom parameters constrained
*S* = 1.02	*w* = 1/[σ^2^(*F*_o_^2^) + (0.083*P*)^2^ + 0.1225*P*] where *P* = (*F*_o_^2^ + 2*F*_c_^2^)/3
6646 reflections	(Δ/σ)_max_ < 0.001
247 parameters	Δρ_max_ = 0.25 e Å^−^^3^
0 restraints	Δρ_min_ = −0.21 e Å^−^^3^

### Special details

**Table:**

Geometry. All e.s.d.'s (except the e.s.d. in the dihedral angle between two l.s. planes) are estimated using the full covariance matrix. The cell e.s.d.'s are taken into account individually in the estimation of e.s.d.'s in distances, angles and torsion angles; correlations between e.s.d.'s in cell parameters are only used when they are defined by crystal symmetry. An approximate (isotropic) treatment of cell e.s.d.'s is used for estimating e.s.d.'s involving l.s. planes.
Refinement. Refinement of *F*^2^ against ALL reflections. The weighted *R*-factor *wR* and goodness of fit *S* are based on *F*^2^, conventional *R*-factors *R* are based on *F*, with *F* set to zero for negative *F*^2^. The threshold expression of *F*^2^ > σ(*F*^2^) is used only for calculating *R*-factors(gt) *etc*. and is not relevant to the choice of reflections for refinement. *R*-factors based on *F*^2^ are statistically about twice as large as those based on *F*, and *R*- factors based on ALL data will be even larger.

### Fractional atomic coordinates and isotropic or equivalent isotropic displacement parameters (Å^2^)

**Table:**

	*x*	*y*	*z*	*U*_iso_*/*U*_eq_	
C1	0.31037 (15)	0.58360 (15)	1.00970 (12)	0.0513 (3)	
H1	0.3275	0.4934	1.0602	0.062*	
C2	0.27416 (17)	0.69925 (18)	1.06433 (12)	0.0587 (4)	
H2	0.2698	0.6871	1.1523	0.070*	
C3	0.24384 (16)	0.83397 (16)	0.99082 (13)	0.0544 (3)	
H3	0.2180	0.9113	1.0298	0.065*	
C4	0.25171 (13)	0.85470 (13)	0.85931 (12)	0.0429 (3)	
C5	0.29508 (12)	0.73676 (12)	0.80276 (10)	0.0370 (2)	
C6	0.32154 (13)	0.60162 (13)	0.87760 (11)	0.0407 (2)	
C7	0.35960 (15)	0.47780 (13)	0.81699 (13)	0.0476 (3)	
H7	0.3812	0.4937	0.7303	0.057*	
C8	0.14625 (18)	1.10162 (16)	0.82610 (18)	0.0674 (4)	
H8A	0.2085	1.1287	0.8779	0.081*	
H8B	0.0645	1.0803	0.8781	0.081*	
C9	0.1000 (2)	1.21793 (18)	0.7157 (2)	0.0875 (6)	
H9A	0.1819	1.2399	0.6664	0.131*	
H9B	0.0477	1.2999	0.7455	0.131*	
H9C	0.0405	1.1890	0.6640	0.131*	
C10	0.41026 (15)	0.83034 (13)	0.61113 (12)	0.0460 (3)	
H10A	0.4742	0.8363	0.6735	0.055*	
H10B	0.3643	0.9254	0.5711	0.055*	
C11	0.49176 (13)	0.75448 (12)	0.51363 (11)	0.0411 (3)	
C12	0.60371 (14)	0.63091 (13)	0.56688 (11)	0.0448 (3)	
C13	0.79009 (18)	0.44609 (17)	0.52593 (16)	0.0675 (4)	
H13A	0.8616	0.4799	0.5595	0.101*	
H13B	0.8308	0.4006	0.4575	0.101*	
H13C	0.7542	0.3799	0.5915	0.101*	
C14	0.47471 (13)	0.79509 (12)	0.38798 (11)	0.0411 (3)	
H14	0.5362	0.7412	0.3376	0.049*	
C15	0.37025 (13)	0.91476 (12)	0.32064 (11)	0.0403 (3)	
C16	0.41376 (13)	0.99223 (13)	0.20705 (10)	0.0415 (3)	
H16	0.5049	0.9635	0.1739	0.050*	
C17	0.32363 (13)	1.11147 (13)	0.14246 (11)	0.0436 (3)	
H17	0.3567	1.1638	0.0687	0.052*	
C18	0.18579 (14)	1.15383 (13)	0.18558 (12)	0.0438 (3)	
C19	0.13937 (15)	1.07222 (15)	0.29530 (15)	0.0588 (4)	
H19	0.0455	1.0962	0.3239	0.071*	
C20	0.23041 (15)	0.95586 (15)	0.36287 (14)	0.0562 (4)	
H20	0.1976	0.9046	0.4374	0.067*	
C21	0.08788 (16)	1.28446 (14)	0.11572 (14)	0.0529 (3)	
H21A	0.1371	1.3239	0.0396	0.064*	
H21B	0.0058	1.2580	0.0898	0.064*	
C22	0.03783 (18)	1.39611 (16)	0.19423 (17)	0.0664 (4)	
H22A	−0.0149	1.3593	0.2678	0.100*	
H22B	−0.0219	1.4771	0.1445	0.100*	
H22C	0.1185	1.4229	0.2203	0.100*	
O1	0.36468 (14)	0.35836 (11)	0.87125 (11)	0.0706 (3)	
O2	0.21967 (11)	0.98154 (9)	0.77707 (9)	0.0546 (3)	
O3	0.30428 (9)	0.75349 (9)	0.67258 (7)	0.0420 (2)	
O4	0.62780 (13)	0.59403 (13)	0.67774 (9)	0.0718 (3)	
O5	0.67596 (12)	0.56277 (10)	0.47986 (9)	0.0596 (3)	

### Atomic displacement parameters (Å^2^)

**Table:**

	*U*^11^	*U*^22^	*U*^33^	*U*^12^	*U*^13^	*U*^23^
C1	0.0547 (8)	0.0589 (8)	0.0368 (6)	−0.0115 (6)	−0.0013 (5)	−0.0034 (5)
C2	0.0677 (9)	0.0775 (10)	0.0329 (6)	−0.0189 (7)	0.0017 (6)	−0.0135 (6)
C3	0.0581 (8)	0.0663 (8)	0.0470 (7)	−0.0161 (6)	0.0069 (6)	−0.0294 (6)
C4	0.0428 (6)	0.0439 (6)	0.0434 (6)	−0.0083 (5)	0.0031 (5)	−0.0150 (5)
C5	0.0374 (6)	0.0427 (6)	0.0313 (5)	−0.0076 (4)	0.0004 (4)	−0.0103 (4)
C6	0.0399 (6)	0.0442 (6)	0.0367 (5)	−0.0080 (4)	0.0002 (4)	−0.0072 (4)
C7	0.0534 (7)	0.0417 (6)	0.0442 (6)	−0.0067 (5)	0.0025 (5)	−0.0065 (5)
C8	0.0644 (10)	0.0504 (8)	0.0862 (11)	0.0030 (7)	0.0051 (8)	−0.0307 (8)
C9	0.0839 (13)	0.0477 (9)	0.1221 (17)	0.0050 (8)	−0.0087 (12)	−0.0170 (9)
C10	0.0620 (8)	0.0391 (6)	0.0384 (6)	−0.0151 (5)	0.0069 (5)	−0.0099 (4)
C11	0.0490 (7)	0.0375 (5)	0.0353 (5)	−0.0085 (5)	0.0015 (5)	−0.0061 (4)
C12	0.0506 (7)	0.0459 (6)	0.0354 (6)	−0.0084 (5)	−0.0033 (5)	−0.0043 (5)
C13	0.0659 (10)	0.0592 (9)	0.0648 (9)	0.0140 (7)	−0.0140 (7)	−0.0106 (7)
C14	0.0472 (6)	0.0359 (5)	0.0366 (5)	−0.0041 (4)	0.0007 (5)	−0.0059 (4)
C15	0.0462 (6)	0.0352 (5)	0.0372 (5)	−0.0057 (4)	−0.0009 (5)	−0.0061 (4)
C16	0.0410 (6)	0.0471 (6)	0.0335 (5)	−0.0054 (5)	−0.0007 (4)	−0.0065 (4)
C17	0.0480 (7)	0.0451 (6)	0.0351 (5)	−0.0096 (5)	−0.0041 (5)	−0.0021 (4)
C18	0.0460 (7)	0.0378 (6)	0.0463 (6)	−0.0065 (5)	−0.0064 (5)	−0.0064 (5)
C19	0.0440 (7)	0.0520 (7)	0.0676 (9)	−0.0015 (5)	0.0100 (6)	0.0019 (6)
C20	0.0522 (8)	0.0474 (7)	0.0569 (8)	−0.0064 (6)	0.0117 (6)	0.0063 (6)
C21	0.0512 (7)	0.0462 (7)	0.0557 (8)	−0.0027 (5)	−0.0115 (6)	−0.0029 (6)
C22	0.0629 (9)	0.0505 (8)	0.0788 (10)	0.0062 (7)	−0.0115 (8)	−0.0145 (7)
O1	0.0969 (9)	0.0419 (5)	0.0657 (7)	−0.0109 (5)	0.0071 (6)	−0.0030 (5)
O2	0.0641 (6)	0.0391 (5)	0.0563 (5)	−0.0010 (4)	0.0073 (5)	−0.0149 (4)
O3	0.0526 (5)	0.0433 (4)	0.0317 (4)	−0.0127 (3)	0.0005 (3)	−0.0090 (3)
O4	0.0754 (8)	0.0890 (8)	0.0372 (5)	0.0058 (6)	−0.0124 (5)	−0.0039 (5)
O5	0.0704 (7)	0.0530 (5)	0.0423 (5)	0.0145 (5)	−0.0087 (4)	−0.0085 (4)

### Geometric parameters (Å, °)

**Table:**

C1—C2	1.366 (2)	C11—C12	1.4829 (17)
C1—C6	1.3969 (17)	C12—O4	1.1990 (15)
C1—H1	0.9300	C12—O5	1.3347 (16)
C2—C3	1.382 (2)	C13—O5	1.4370 (16)
C2—H2	0.9300	C13—H13A	0.9600
C3—C4	1.3882 (18)	C13—H13B	0.9600
C3—H3	0.9300	C13—H13C	0.9600
C4—O2	1.3632 (15)	C14—C15	1.4696 (15)
C4—C5	1.4019 (16)	C14—H14	0.9300
C5—O3	1.3771 (13)	C15—C20	1.3905 (18)
C5—C6	1.3901 (16)	C15—C16	1.3908 (16)
C6—C7	1.4763 (17)	C16—C17	1.3856 (16)
C7—O1	1.1978 (16)	C16—H16	0.9300
C7—H7	0.9300	C17—C18	1.3785 (18)
C8—O2	1.4259 (16)	C17—H17	0.9300
C8—C9	1.488 (3)	C18—C19	1.3901 (19)
C8—H8A	0.9700	C18—C21	1.5106 (17)
C8—H8B	0.9700	C19—C20	1.3853 (19)
C9—H9A	0.9600	C19—H19	0.9300
C9—H9B	0.9600	C20—H20	0.9300
C9—H9C	0.9600	C21—C22	1.509 (2)
C10—O3	1.4520 (15)	C21—H21A	0.9700
C10—C11	1.4949 (16)	C21—H21B	0.9700
C10—H10A	0.9700	C22—H22A	0.9600
C10—H10B	0.9700	C22—H22B	0.9600
C11—C14	1.3414 (16)	C22—H22C	0.9600
			
C2—C1—C6	119.61 (12)	O5—C12—C11	113.84 (10)
C2—C1—H1	120.2	O5—C13—H13A	109.5
C6—C1—H1	120.2	O5—C13—H13B	109.5
C1—C2—C3	121.00 (12)	H13A—C13—H13B	109.5
C1—C2—H2	119.5	O5—C13—H13C	109.5
C3—C2—H2	119.5	H13A—C13—H13C	109.5
C2—C3—C4	120.52 (12)	H13B—C13—H13C	109.5
C2—C3—H3	119.7	C11—C14—C15	127.62 (11)
C4—C3—H3	119.7	C11—C14—H14	116.2
O2—C4—C3	125.90 (11)	C15—C14—H14	116.2
O2—C4—C5	115.38 (10)	C20—C15—C16	117.66 (11)
C3—C4—C5	118.72 (12)	C20—C15—C14	124.35 (11)
O3—C5—C6	119.47 (10)	C16—C15—C14	117.99 (11)
O3—C5—C4	120.26 (10)	C17—C16—C15	121.10 (11)
C6—C5—C4	120.17 (10)	C17—C16—H16	119.4
C5—C6—C1	119.87 (11)	C15—C16—H16	119.4
C5—C6—C7	119.81 (10)	C18—C17—C16	121.16 (11)
C1—C6—C7	120.32 (11)	C18—C17—H17	119.4
O1—C7—C6	124.82 (12)	C16—C17—H17	119.4
O1—C7—H7	117.6	C17—C18—C19	117.90 (11)
C6—C7—H7	117.6	C17—C18—C21	121.17 (11)
O2—C8—C9	107.34 (15)	C19—C18—C21	120.94 (12)
O2—C8—H8A	110.2	C20—C19—C18	121.21 (13)
C9—C8—H8A	110.2	C20—C19—H19	119.4
O2—C8—H8B	110.2	C18—C19—H19	119.4
C9—C8—H8B	110.2	C19—C20—C15	120.81 (12)
H8A—C8—H8B	108.5	C19—C20—H20	119.6
C8—C9—H9A	109.5	C15—C20—H20	119.6
C8—C9—H9B	109.5	C22—C21—C18	113.21 (12)
H9A—C9—H9B	109.5	C22—C21—H21A	108.9
C8—C9—H9C	109.5	C18—C21—H21A	108.9
H9A—C9—H9C	109.5	C22—C21—H21B	108.9
H9B—C9—H9C	109.5	C18—C21—H21B	108.9
O3—C10—C11	108.76 (10)	H21A—C21—H21B	107.7
O3—C10—H10A	109.9	C21—C22—H22A	109.5
C11—C10—H10A	109.9	C21—C22—H22B	109.5
O3—C10—H10B	109.9	H22A—C22—H22B	109.5
C11—C10—H10B	109.9	C21—C22—H22C	109.5
H10A—C10—H10B	108.3	H22A—C22—H22C	109.5
C14—C11—C12	120.99 (11)	H22B—C22—H22C	109.5
C14—C11—C10	125.04 (11)	C4—O2—C8	118.22 (11)
C12—C11—C10	113.86 (10)	C5—O3—C10	114.56 (9)
O4—C12—O5	122.44 (12)	C12—O5—C13	115.99 (11)
O4—C12—C11	123.71 (12)		
			
C6—C1—C2—C3	2.1 (2)	C10—C11—C14—C15	−3.6 (2)
C1—C2—C3—C4	−1.0 (2)	C11—C14—C15—C20	−38.7 (2)
C2—C3—C4—O2	177.56 (13)	C11—C14—C15—C16	141.38 (14)
C2—C3—C4—C5	−1.9 (2)	C20—C15—C16—C17	4.00 (19)
O2—C4—C5—O3	0.58 (17)	C14—C15—C16—C17	−176.03 (11)
C3—C4—C5—O3	−179.90 (11)	C15—C16—C17—C18	−2.89 (19)
O2—C4—C5—C6	−175.87 (11)	C16—C17—C18—C19	−0.7 (2)
C3—C4—C5—C6	3.65 (18)	C16—C17—C18—C21	179.52 (12)
O3—C5—C6—C1	−179.04 (11)	C17—C18—C19—C20	3.1 (2)
C4—C5—C6—C1	−2.56 (18)	C21—C18—C19—C20	−177.14 (14)
O3—C5—C6—C7	0.21 (18)	C18—C19—C20—C15	−1.9 (3)
C4—C5—C6—C7	176.69 (11)	C16—C15—C20—C19	−1.6 (2)
C2—C1—C6—C5	−0.3 (2)	C14—C15—C20—C19	178.41 (14)
C2—C1—C6—C7	−179.58 (13)	C17—C18—C21—C22	−118.36 (15)
C5—C6—C7—O1	−168.90 (14)	C19—C18—C21—C22	61.87 (19)
C1—C6—C7—O1	10.3 (2)	C3—C4—O2—C8	−12.8 (2)
O3—C10—C11—C14	103.91 (14)	C5—C4—O2—C8	166.63 (12)
O3—C10—C11—C12	−79.81 (13)	C9—C8—O2—C4	−169.18 (14)
C14—C11—C12—O4	176.44 (14)	C6—C5—O3—C10	−119.28 (12)
C10—C11—C12—O4	0.0 (2)	C4—C5—O3—C10	64.25 (14)
C14—C11—C12—O5	−3.89 (18)	C11—C10—O3—C5	135.17 (10)
C10—C11—C12—O5	179.67 (11)	O4—C12—O5—C13	−3.1 (2)
C12—C11—C14—C15	−179.59 (12)	C11—C12—O5—C13	177.20 (13)

### Hydrogen-bond geometry (Å, °)

**Table:**

*D*—H···*A*	*D*—H	H···*A*	*D*···*A*	*D*—H···*A*
C7—H7···O5^i^	0.93	2.58	3.368 (2)	143
C17—H17···O1^ii^	0.93	2.56	3.454 (2)	161

Symmetry codes: (i) −*x*+1, −*y*+1, −*z*+1; (ii) *x*, *y*+1, *z*−1.
